# Supplementary material for: Resveratrol and (-)-Epigallocatechin-3-gallate Regulate Lipid Metabolism by Activating the AMPK Pathway in Hepatocytes
Source: Biology (Basel). 2024 May 23;13(6):368. doi: 10.3390/biology13060368 (PMC11201192; doi:10.3390/biology13060368)
Supplement: Supplementary file 1 [file biology-13-00368-s001.zip › biology-3010426-supplementary.pdf]

Supplementary materials

AMPK (n=3):

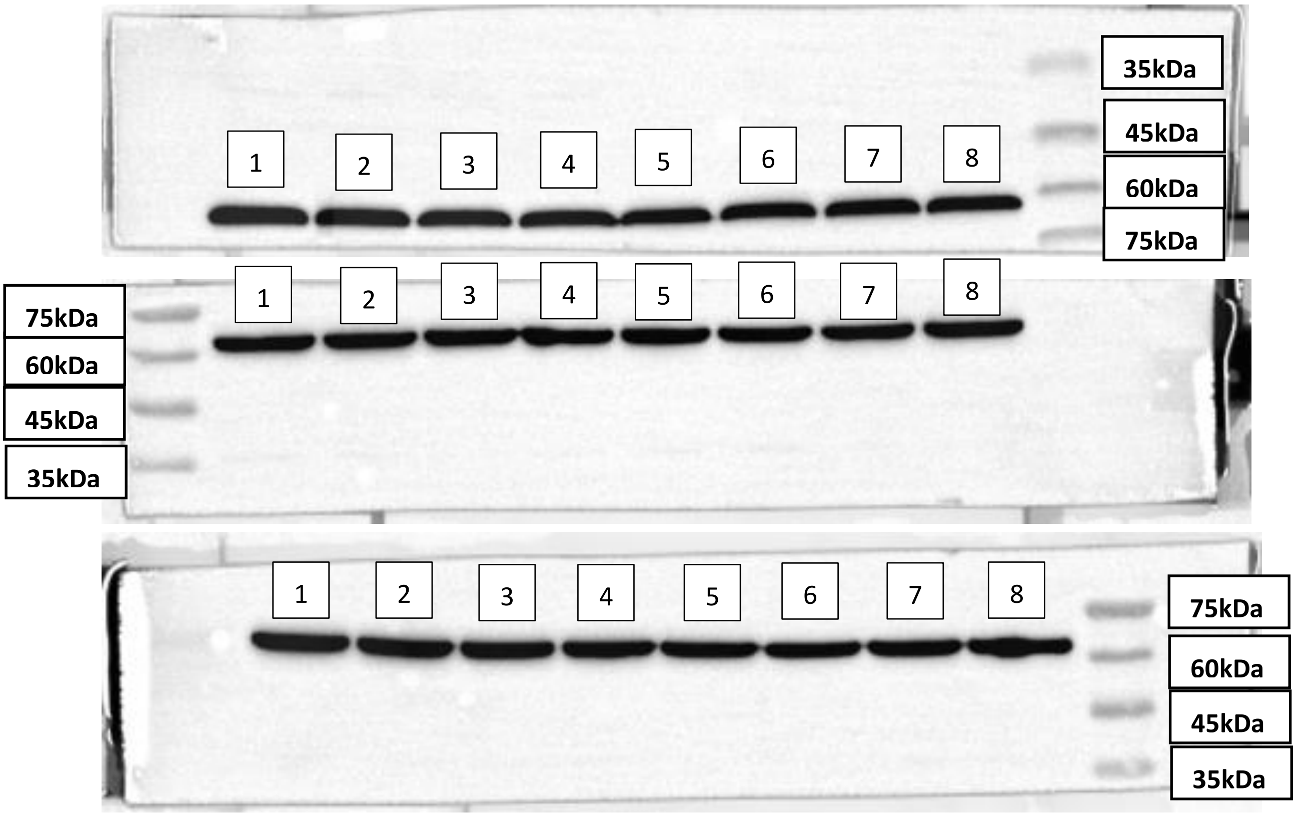

P-AMPK (n=3):

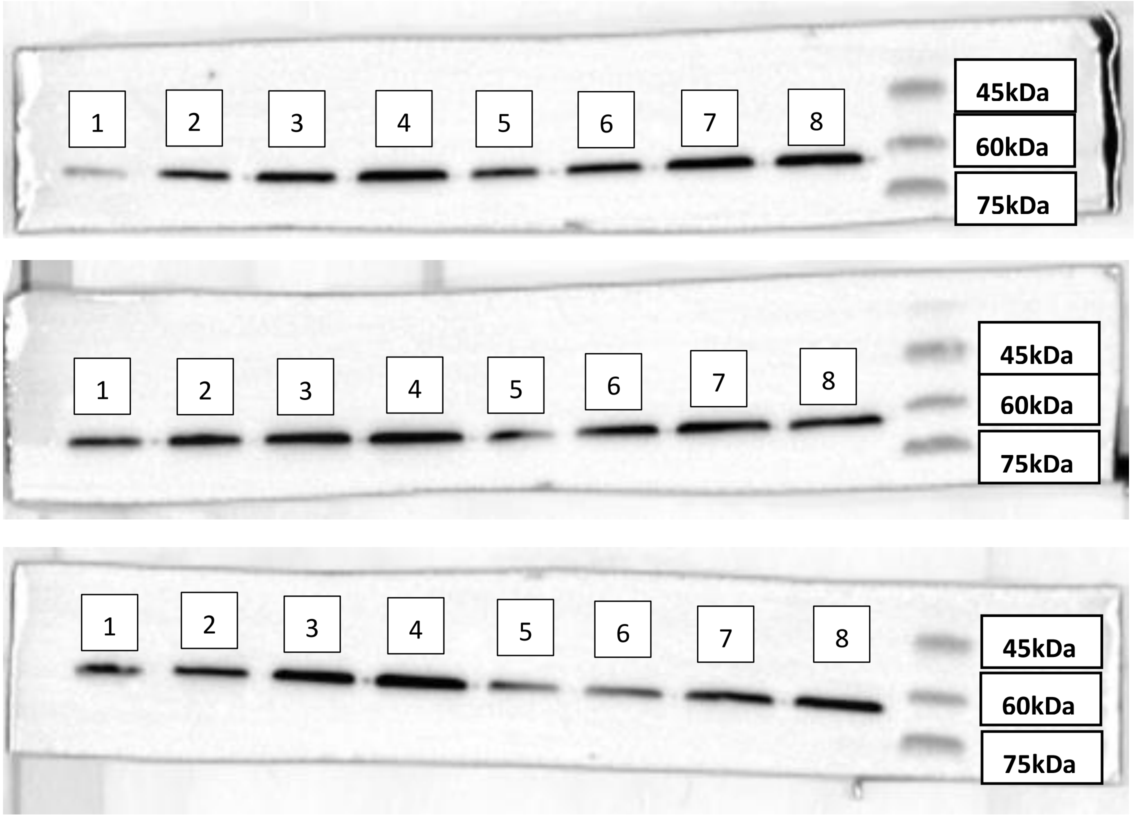

GAPDH (n=3):

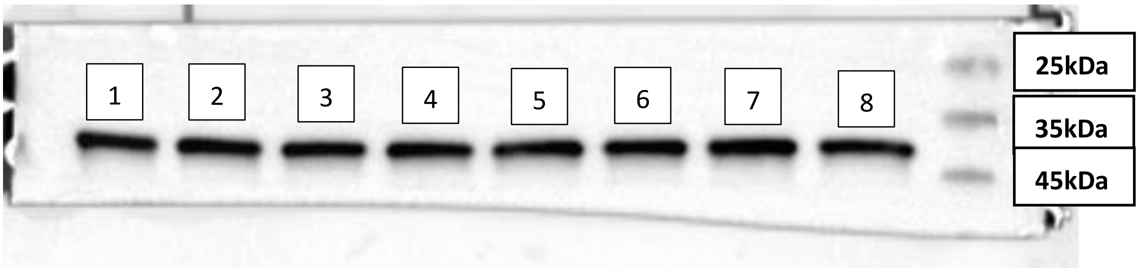

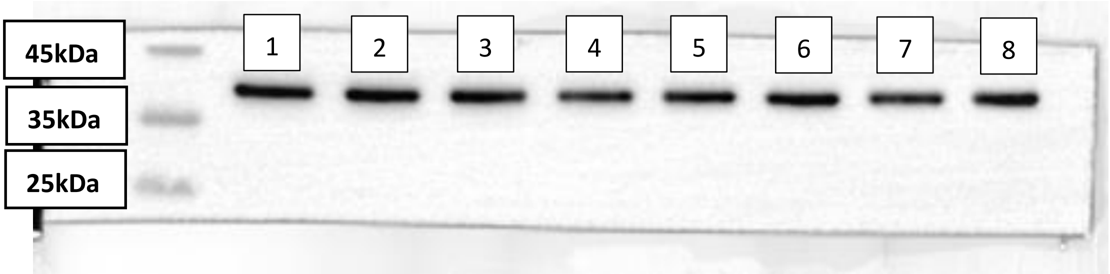

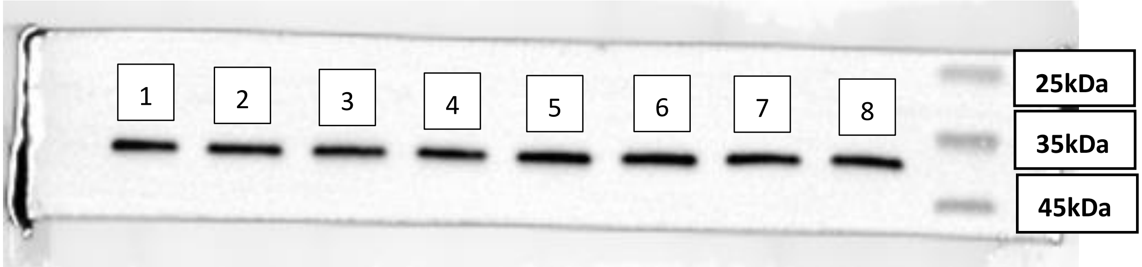

HMGCR (n=3):

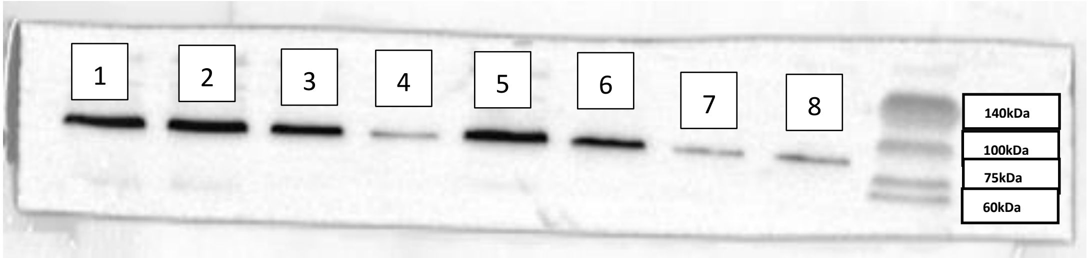

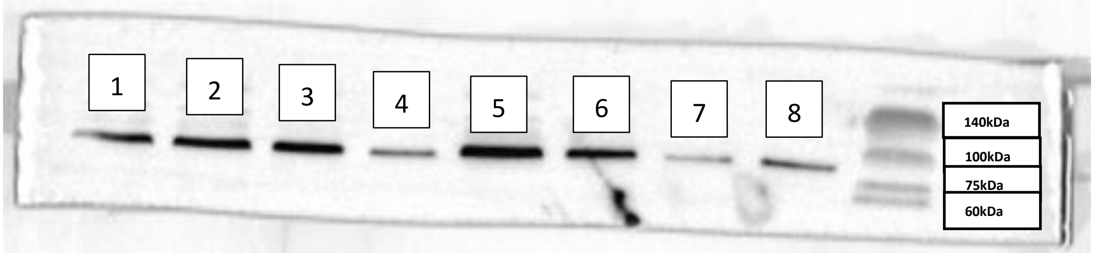

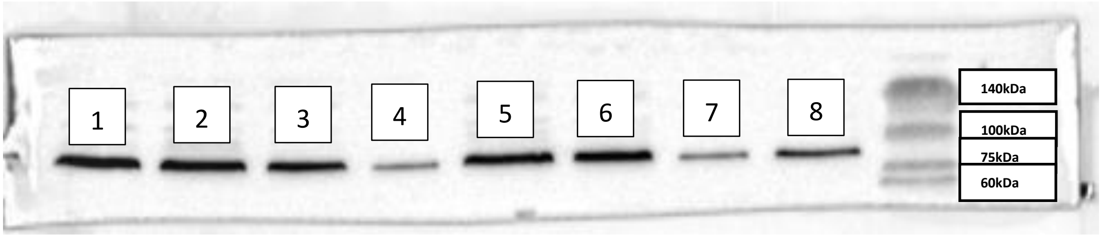

GAPDH (n=3):

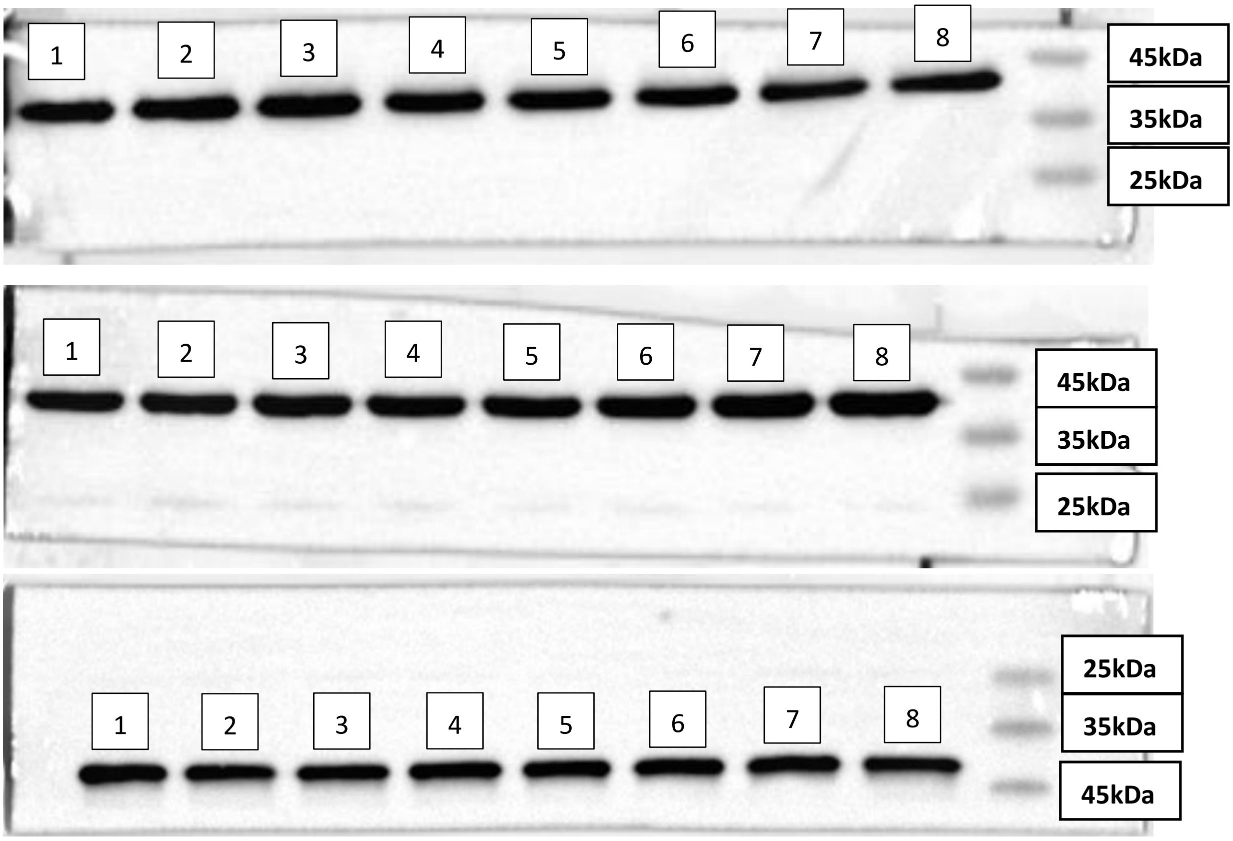

Figure S1. Protein blot experiment results. 1: control group; 2: 6.25 μM Res group; 3: 12.5 μM Res group; 4: 25 μM Res group; 5: 6.25 μM EGCG group; 6: 12.5 μM EGCG group; 7: 25 μM EGCG group; 8: Composite group.
